# High detection rate of dog circovirus in diarrheal dogs

**DOI:** 10.1186/s12917-016-0722-8

**Published:** 2016-06-17

**Authors:** Han-Siang Hsu, Ting-Han Lin, Hung-Yi Wu, Lee-Shuan Lin, Cheng-Shu Chung, Ming-Tang Chiou, Chao-Nan Lin

**Affiliations:** Department of Veterinary Medicine, College of Veterinary Medicine, National Pingtung University of Science and Technology, Neipu, Pingtung, Taiwan; Graduate Institute of Veterinary Pathobiology, College of Veterinary Medicine, National Chung-Hsing University, South Dist, Taichung, Taiwan; Veterinary Hospital, College of Veterinary Medicine, National Pingtung University of Science and Technology, Neipu, Pingtung, Taiwan; Animal Disease Diagnostic Center, College of Veterinary Medicine, National Pingtung University of Science and Technology, Neipu, Pingtung, Taiwan

**Keywords:** Dog circovirus, Diarrhea, DogCV, Real-time PCR

## Abstract

**Background:**

Diarrhea is one of the most common clinical symptoms reported in companion animal clinics. Dog circovirus (DogCV) is a new mammalian circovirus that is considered to be a cause of alimentary syndromes such as diarrhea, vomiting and hemorrhagic enteritis. DogCV has previously only been identified in the United States, Italy, Germany (GeneBank accession number: KF887949) and China (GeneBank accession number: KT946839). Therefore, the aims of this study were to determine the prevalence of DogCV in Taiwan and to explore the correlation between diarrhea and DogCV infection. Clinical specimens were collected between 2012 and 2014 from 207 dogs suffering from diarrhea and 160 healthy dogs.

**Results:**

In this study, we developed a sensitive and specific SYBR Green-based real-time PCR assays to detected DogCV in naturally infected animals. Of the analyzed fecal samples from diarrheal dogs and health dogs, 58 (28.0 %) and 19 (11.9 %), respectively, were DogCV positive. The difference in DogCV prevalence was highly significant (*P* = 0.0002755) in diarrheal dogs.

**Conclusions:**

This is the first study to reveal that DogCV is currently circulating in domestic dogs in Taiwan and to demonstrate its high detection rate in dogs with diarrhea.

**Electronic supplementary material:**

The online version of this article (doi:10.1186/s12917-016-0722-8) contains supplementary material, which is available to authorized users.

## Background

Gastrointestinal disorders are one of the most common diseases reported in companion animal clinics. They can be caused by a number of viral, bacterial and parasitic pathogens. The most common viral gastrointestinal-pathogens are canine parvovirus [1, 2] and coronavirus. However, other agents, such as dog circovirus (DogCV), have recently been considered to be related to enteric disorders in dogs [3, 4]. DogCV was first identified in dogs with vasculitis and/or hemorrhagic gastroenteritis in the United States in 2012 [4]. DogCV is a non-enveloped, circular, single-stranded DNA virus containing a circular genome approximately 2 kb in length. It belongs to the genus *Circovirus*, together with porcine circovirus type 1 (PCV1), porcine circovirus type 2 (PCV2), canary circovirus, beak and feather disease virus and other viruses of domestic and wild birds [3].

PCV2 causes clinical conditions including systemic, lung, enteric, reproductive and skin diseases [5]. In recent years, a possible association between DogCV and canine enteritis has been suggested [3, 4]. DogCV has also been reported to cause necrotizing lymphadenitis [4] and vasculitis, which are also caused by porcine circovirus type 2 infections in pigs [5]. Previous studies have shown that DogCV is associated with hemorrhagic enteritis in dogs [3, 4], however, limited information is available to determine the direct correlation between the severity of diarrhea and DogCV infections.

DogCV has only been detected in the US [4, 6], Italy [3], Germany (GeneBank accession number: KF887949) and China (GeneBank accession number: KT946839). In the present study, we determined the previously unidentified DogCV and its prevalence in Taiwanese household dogs and clarified the correlation between diarrhea and DogCV infection.

## Methods

### Ethics and consents

The study did not involve any animal experiment. The Institutional Animal Care and Use Committee (IACUC) of National Pingtung University of Science and Technology did not deem it necessary for this research group to obtain formal approval to conduct this study. The dog owners gave his/her written consent for sample collection and data publication. Besides, according to Dr. Baneux’ recommendations “Privately owned animals that are recruited for clinical studies (not Public Health Service funded) do not need to be subjected to IACUC oversight as long as their involvement includes only procedures that are consistent with the standard of care provided to patients with the same diagnosis that are not included in the clinical study” [7].

### Specimen collection and DNA extraction

Clinical specimens, including rectal swabs or feces, were collected from dogs presenting at animal hospitals in Taiwan between June 2012 and June 2014. A total of 367 household dogs were enrolled in this study. These included 160 healthy dogs with no symptoms of diarrhea (for routine health check) and 207 dogs with diarrhea. The age, breed, sex of each dog and the date of sampling were recorded. The range of age span for dogs with and without diarrhea was from 2 months - 13 years old and 2.5 months - 18 years old, respectively. Fecal scores were determined and recorded according to the Waltham Fecal Scoring System. For detection of DogCV, fecal samples from these dogs with and without diarrhea were collected by inserting the swab into the rectum (~2 cm), rotating the swab and then placing the swab in the nuclease-free water. To ensure the diarrhea only results from DogCV infection, fecal samples taken from diarrheal dogs were also tested for CPV-2, CCV, giardia (CPV/CCV/Giardia Ag Test Kit, BIONOTE, Republic of Korea) and CDV (CDV Ag Test Kit, BIONOTE, Republic of Korea). All clinical specimens were sent to the animal disease diagnostic center at the National Pingtung University of Science and Technology. The weight of the stool sample was diluted 1:10 (w/v) with PBS, and vortexed for 5 min at maximum speed to generate a stool suspension. Viral DNA was extracted from clinical samples using the Genomic DNA Mini Kit (Geneaid Biotech, Ltd., Taipei, Taiwan) according to the manufacturer’s protocol. GAPDH was used for DNA quality control of all samples. All of specimens were screened using real-time PCR for CPV-2, as described by Lin et al [8] and for DogCV, as described below.

### Primer design for DogCV

**Fig. 1:**
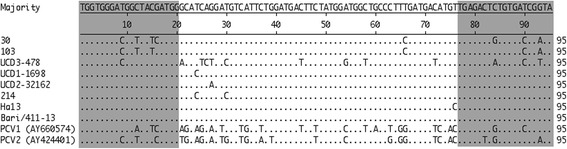
Nucleotide sequence alignment of the 95-bp fragments of the replicase gene from Dog circovirus, porcine circovirus type 1 and porcine circovirus type 2. Only nucleotides differing from the overall majority sequence are shown in uppercase. The primer set is shown in light gray

Based on the sequence homology of PCV1, PCV2 and DogCV, a conserved region of the replicase gene of PCV1, PCV2 and DogCV was identified in nucleotide sequences available from GenBank (PCV1: AY660574; PCV2: AY424401; DogCV: JQ821392, NC020904, KC241983, KC241984, KF887949, [9] KJ530972 and KT946839). The sequences were aligned with the Clustal W method using the MegAlign program (DNASTAR, Madison, WI). Figure 1 shows the nucleotide sequence alignment of the partial replicase genes of PCV1, PCV2 and DogCVs.

### Construction of plasmid DNA standard curves

The oligonucleotide primers used for the amplification of PCV1, PCV2 and DogCV were Rep CircoV-F, 5’-TGG TGG GAY GGH TAY SAT GG-3’ and Rep CircoV-R, 5’-TAH CGR TCA CAB ART CTC A-3’; the sequences of these primers correspond to base pair positions 607-626 and 701-683, respectively, of DogCV strain NY214 (GenBank accession number JQ821932) [6]. Using these primers, 95 bp fragments of the replicase gene were amplified from PCV1, PCV2 and DogCV. The PCR products were cloned using the TA cloning kit (Yeastern Biotech Co., Ltd. Taipei, Taiwan) and sequenced. The plasmids containing the circovirus sequences were purified using a plasmid miniprep purification kit (GMbiolab Co, Ltd. Taichung, Taiwan) and quantified by the measurement of optical density (OD) at 260 nm with a spectrophotometer (Hitachi U2900, Dallas, TX, USA). A standard curve was generated using 10-fold dilutions (10^2^-10^8^ copies/μl) of standard plasmid DNA to determine the detection limits of the SYBR Green-based real-time PCR assay. Intra- (within-run) and inter- (between-run) assay reproducibility was evaluated using the standard plasmid DNA in triplicate on three different days.

### Real-time PCR for the detection of DogCV

SYBR Green-based real-time PCR assays were performed using a LightCycler Nano (Roche Diagnostics, Mannheim, Germany). Each 10-μl reaction mixture contained 0.2 μM of forward and reverse primers, 5 μl of Master buffer (Roche Diagnostics, Mannheim, Germany) and 3 μl of DNA extract. The thermocycling conditions consisted of 10 min at 95 °C, 45 cycles of 10 s each at 95 °C, 10 s at 51 °C and 10 s at 72 °C. Each run included serial 10-fold dilutions of the standard plasmid DNA as a positive control and for the construction of a standard curve. A negative control without the DNA template was included to detect possible cross-contamination.

### Replicase gene sequencing and sequence analysis

The DNA fragments were purified and sequenced, as described by Lin et al [10]. The partial replicase DNA sequence of our samples were compared to those of reference DogCV (JQ821392, NC020904, KC241983, KC241984, KF887949 and KJ530972), PCV1 (AY660574) and PCV2 (AY424401). Multiple alignments of the nucleic acid sequences were performed with the Clustal W method, using the MegAlign program (DNASTAR, Madison, WI).

### Statistical analysis

The chi-square test with Yate’s correction was used to evaluate the correlation between the samples that were positive for DogCV and different clinical observation groups. The paired *t*-test was used to determine whether there is a statistically significant difference in age between diarrheal and healthy groups. A Wilcoxon Rank-Sum test was used to evaluate the correlation between the severity of diarrhea and samples positive for DogCV. Differences were considered to be statistically significant and highly significant if the associated P-value was <0.05 and <0.01, respectively.

## Results

### Real-time PCR amplification efficiency and limits of detection

The optimized PCR assay targeting a conserved region of the replicase gene permitted the detection of PCV1, PCV2 and DogCV (data not shown). The standard curve generated using standard plasmid DNA had a linear range of seven orders of magnitude (10^2^ to 10^8^ copies/μl). The correlation coefficient (*R*^*2*^) between the number of quantification cycles (Cq) and the logarithm of the plasmid copy number was 0.999 (slope = -4.18), based on triplicate runs (data not shown). The average *T*m value of DogCV was approximately 80 °C (data not shown). To assess the detection limits of the SYBR Green-based real-time PCR assays, standard plasmid DNA was serially diluted 10-fold ranging from 10^2^ to 10^8^ copies/μl, and was used as a template. At 10^3^ copies per reaction, 100 % of the replicates were found to be positive using real-time PCR, whereas only 90 % of the replicates were positive at 10^2^ copies (Table 1). The coefficients of variation of the within-run and between-run mean Cq values for standard plasmid DNA ranged from 0.41 to 1.33 % and from 2.45 to 6.84 %, respectively (data not shown). To test the specificity of the SYBR Green-based real-time PCR assay, we analyzed canine distemper virus (CDV), canine coronavirus (CCV) and canine parvovirus type 2 (CPV-2). No specific amplification was detected for any of these samples (data not shown).

**Table 1:** Efficiency of the Dog circovirus (DogCV) SYBR Green-based real-time PCR assay

Estimated number of DogCV plasmid DNA copies	Number of positive/number of tested	Mean Cq ± SD
10^8^	10/10	14.64 ± 0.62
10^7^	10/10	18.75 ± 0.83
10^6^	10/10	23.06 ± 1.24
10^5^	10/10	27.61 ± 1.53
10^4^	10/10	32.11 ± 1.71
10^3^	10/10	36.58 ± 2.28
10^2^	9/10	40.19 ± 1.29

### Overall detection of gastrointestinal pathogens using commercial kits and real-time PCR

Between June 2012 and June 2014, a total of 207 specimens were obtained from 207 enrolled dogs. Of these 207 cases, 74 (35.7 %) were positive for at least one gastrointestinal pathogen, as determined by a commercial kit and real-time PCR. Among those dogs with positive results, most were positive for DogCV (58/74, 78.4 %), followed by CPV-2 (22/74, 29.7 %), giardia (8/74, 10.8 %), CCV (4/74, 5.4 %) and CDV (3/74, 4.1 %). The correlations between the severity of diarrhea and positive tests for the different canine gastrointestinal pathogens are summarized in Table 2.

**Table 2:** Correlation between the severity of diarrhea and the different canine gastrointestinal pathogens

Pathogen	The Waltham fecal scoring system				Total
	5	4.5	4	3.5	
DogCV only	15	11	16	1	43
DogCV + other pathogens	14	1	0	0	15
Other pathogens (without DogCV)	12	3	1	0	16
All negative	30	50	51	2	133
Total	71	65	68	3	207

### Correlation of diarrhea with positive test for DogCV

To understand the prevalence of DogCV in clinically healthy dogs, DogCV was also screened for in fecal samples from 160 healthy dogs using real-time PCR. Nineteen (11.9 %) samples were positive for DogCV. Using a chi-square test, the correlation of the diarrhea with a positive test for DogCV was calculated. Surprisingly, among the 367 dogs analyzed, a positive DogCV test had a highly significant correlation with the presence of diarrhea in dogs (*P* = 0.0002755)(Table 3). The odds for diarrheal and healthy groups were 0.39 and 0.13, respectively (Table 3), indicating that dogs with diarrhea were 3 times more likely to be DogCV-positive than those which are clinically healthy. In addition, we evaluated the correlation between the severity of diarrhea and a positive test for DogCV. According to the Waltham Fecal Scoring System and the results of the DogCV tests, the difference in the prevalence of DogCV was not significant between different severities of diarrhea. (*P* = 0.2286, Wilcoxon Rank-Sum test).

**Table 3:** Correlation of diarrhea with dog circovirus (DogCV) infection

Clinical status	Results of DogCV detection		Odds	Total
	Negative	Positive		
Diarrheal	149 (51.4 %)	58 (75.3 %)	0.39	207
Healthy	141 (48.6 %)	19 (24.7 %)	0.13	160
Total	290	77		367

### Correlation of age with positive test for DogCV

Of the 58 DogCV-positive dogs in diarrheal group, 25 (43.1 %) were less than 1 year old, 25 (43.1 %) were between 1-7 years old, and 8 (13.8 %) were more than 7 years old. The range of age spans for DogCV-positive dogs was from 2 months - 13 years old (median is 1 year old). Of 19 DogCV-positive dogs in the health group, 3 (15.8 %) were less than 1 year old, 8 (42.1 %) were between 1-7 years old, and 8 (42.1 %) were more than 7 years old. The range of age spans for DogCV-positive dog was from 3 months - 18 years old (median is 5 year old). By statistic analysis, it was found that there was a statistically significant difference in age between diarrheal and healthy groups with respect to the DogCV-positive dogs (*P* = 0.005). That is, the DogCV was more frequently identified from younger dogs in diarrheal group.

### Genetic comparison of the DogCVs

Comparison of the partial replicase nucleotide sequences revealed 97.9 % and 82.1-93.7 % homology within the analyzed local DogCV isolates and between the local DogCV and reference strains, respectively (Table 4). The same nucleotide sequences for local DogCV strains also exhibited 91.6-93.7 % homology with those for Italian DogCV strain Bari/411-13 (Table 4).

**Table 4:** Sequence homology between Taiwan and reference DogCVs

Strains								Strains (Country)
103	Bari/411-13	UCD1-1698	UCD2-32162	Ha13	NY214	UCD3-478	JZ98/2014	
97.9	91.6	90.5	90.5	90.5	89.5	84.2	82.1	30 (Taiwan)
	93.7	92.6	92.6	92.6	91.6	85.3	83.2	103 (Taiwan)
		98.9	98.9	98.9	97.9	86.3	87.4	Bari/411-13 (Italy)
			97.9	97.9	98.9	85.3	88.4	UCD1-1698 (USA)
				97.9	96.8	86.3	86.3	UCD2-32162 (USA)
					96.8	85.3	88.4	Ha13 (Germany)
						86.3	87.4	NY214 (USA)
							82.1	UCD3-478 (USA)

## Discussion

In the present study we determine the prevalence of DogCV in Taiwan. Besides, our results suggest that the positive test results for DogCV appear to have a highly significant correlation with the presence of diarrhea (*P* = 0.0002755) and that dogs with diarrhea were 3 times more likely to be DogCV-positive than those which are clinically healthy. In contrast, a study conducted in the US using real-time PCR reported that the difference in the prevalence of DogCV between healthy and diarrheal dogs was not significant [4]. These differing results might be due to the following: i) geographic distribution, (i.e., USA vs. Taiwan); or ii) primer design (i.e., the specific primer for DogCV used in the previous study (Additional file 1: Figure S1) vs. the degenerate primer for mammal circovirus in our study). In the present study, we established a SYBR-Green-based PCR assay to detect DogCV. This novel, real-time PCR was specific, sensitive and reliable for the amplification of DogCV. Both intra- and inter-assay CVs were satisfactorily low.

Forty-three out of the 58 (74.1 %) DogCV-positive dogs were found to be positive for DogCV alone. The fecal scoring of those dogs was observed to range from “very moist stool” to “watery diarrhea”. This agrees with US and Italian studies, which showed a similar proportion of diarrheal dogs to be infected by DogCV alone [3, 4]. Diarrheal symptoms caused by circovirus have been observed in PCV2-associated enteritis [5]. DogCV DNA has been detected in several lymphoid tissues, including Peyer’s patches and mesenteric lymph nodes [4]. A moderate depletion of lymphocytes was found in DogCV-infected dogs, which might suggest immunosuppression in DogCV-affected dogs [4]. However, the pathogenesis of DogCV infection remains to be elucidated.

A previous study reported that co-infection with other canine pathogens was found in DogCV-positive dogs with diarrhea [4]. Co-infection with canine gastrointestinal pathogens was also observed in the present study. Surprisingly, twelve of the 15 DogCV-positive diarrheal dogs were also found to be CPV2-positive. Co-infection with DogCV and CPV-2 was not observed in the US or Italian studies [3, 4]. We further investigated the antigenic variants of CPV-2, and only CPV-2a or 2b were found in dogs co-infected with DogCV. This might be because no CPV-2c was found in Taiwan during the study period [10]. In PCV2 infection, the expression of clinical disease in infected-pigs typically involves secondary microbial infections [9, 11]. Therefore, the role of co-infection in the pathogenesis of DogCV will require further investigation.

Our results showed that the partial replicase nucleotide sequences for local DogCV strains exhibited 91.6-93.7 % homology with those for Italian DogCV strain Bari/411-13. However, only a few complete genomes of DogCV are available in GeneBank. The patterns of the genetic evolution of DogCV need to be investigated through continuous surveillance and by analyzing the sequence of the complete genome.

## Conclusions

This is the first study to reveal that DogCV is currently circulating in domestic dogs in Taiwan and to demonstrate its high detection rate in dogs with diarrhea.

## Abbreviations

CCV, canine coronavirus; CDV, canine distemper virus; CPV-2, canine parvovirus type 2; Cq, quantification cycles; DogCV, Dog circovirus (DogCV); OD: optical density; PCR, polymerase chain reaction; PCV1, porcine circovirus type 1; PCV2, porcine circovirus type 2; *T*m, melting temperature.

## Additional file

Additional file 1: Figure S1. Primer sequences alignment of DogCVs. Primer sequences (2 primer sets: Capsid and replicate) were highlighted in yellow (Li et al., 2013. Emerg Infect Dis 19(4): 534-541) [4]. Herein, some of the mismatches were found. (DOCX 802 kb)
