# Supplementary material for: High detection rate of dog circovirus in diarrheal dogs
Source: BMC Vet Res. 2016 Jun 17;12:116. doi: 10.1186/s12917-016-0722-8 (PMC4912760; doi:10.1186/s12917-016-0722-8)
Supplement: Additional file 1: Figure S1. — Primer sequences alignment of DogCVs. Primer sequences (2 primer sets: Capsid and replicate) were highlighted in yellow (Li et al., 2013. Emerg Infect Dis 19(4): 534-541) [4]. Herein, some of the mismatches were found. (DOCX 802 kb) [file 12917_2016_722_MOESM1_ESM.docx]

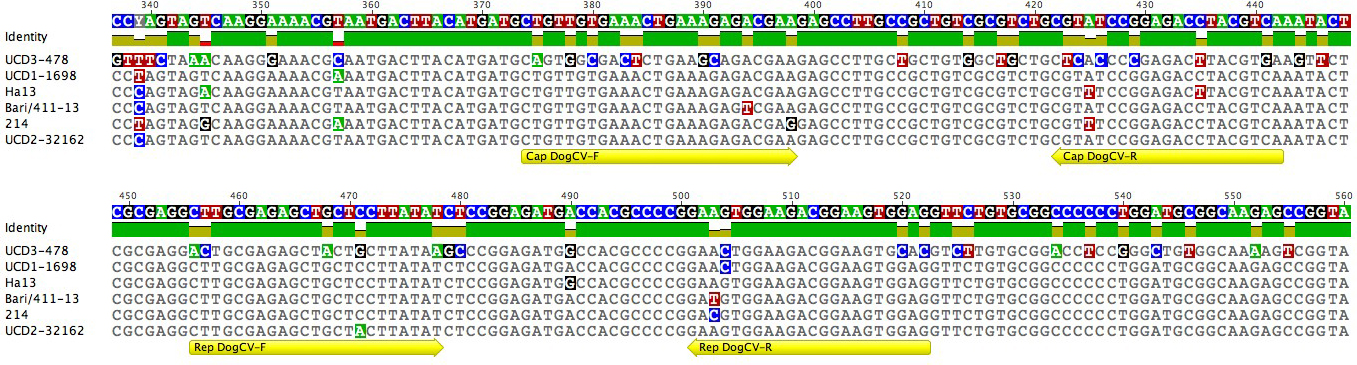


**Supplementary Fig. 1** Primer sequences alignment of DogCVs. Primer sequences (2 primer sets: Capsid and replicate) were highlighted in yellow (Li et al., 2013. Emerg Infect Dis 19(4): 534-541). Herein, some of the mismatches were found.
